# Loop technique-based artificial chordae reconstruction in mitral regurgitation

**DOI:** 10.1007/s00380-025-02618-3

**Published:** 2025-12-22

**Authors:** Takayoshi Kato, Shojiro Yamaguchi, Takatomo Watanabe, Takashi Onuma, Daichi Watanabe, Masayuki Sato, Hiroki Ogura, Etsuji Umeda, Osamu Sakai, Kiyoshi Doi

**Affiliations:** 1 Department of Cardiovascular Surgery, Gifu University Hospital, Gifu University Graduate School of Medicine, 1-1 Yanagido, Gifu, 501-1194 Japan; 2 Department of Cardiology, Gifu University Graduate School of Medicine, Gifu, Japan; 3 Department of Anesthesiology and Pain Medicine, Gifu University Graduate School of Medicine, Gifu, Japan; 4 Department of Pharmacy, Gifu University Hospital, Gifu, Japan; 5 Innovative and Clinical Research Promotion Center, Gifu University Hospital, Gifu, Japan

**Keywords:** Mitral regurgitation, Mitral valve repair, Artificial chordae reconstruction, Loop technique, RAFF method

## Abstract

This study aimed to evaluate the clinical utility of a novel mitral valve repair technique, the Referring to the Anterior, Fixing on the Frontal (RAFF) method, which improves anatomical precision and enhances the reproducibility of loop-type artificial chordae (“neochordae”) reconstruction. This retrospective study included 67 individuals who underwent isolated, elective mitral valve repair with neochordae between 2019 and 2024. Participants were divided into the RAFF (*n* = 36) and non-RAFF (*n* = 31) groups. In the RAFF technique, chordal length is determined by referencing the anterior leaflet chordae from the ipsilateral papillary muscle, with loop sets anchored to the frontal head of the papillary muscle. Leaflet resection was not performed in any case. Baseline demographics and lesion characteristics were similar between groups. The RAFF group demonstrated significantly less residual mitral regurgitation at the conclusion of surgery (*p* = 0.014). Use of the RAFF method was significantly associated with suppression of postoperative leaflet billowing and a lower recurrence rate of moderate or greater mitral regurgitation during follow-up. Frontal fixation of posterior leaflet neochordae, a defining feature of the technique, resulted in a significant increase in coaptation length without inducing systolic anterior motion or elevating transvalvular gradients. The method also significantly reduced aortic cross-clamp time without compromising hemodynamic performance. The RAFF technique offers a standardized and anatomically guided approach to neochordae reconstruction. It minimizes inter-surgeon variability and anatomical inconsistencies, and promotes durable mitral competence by optimizing leaflet coaptation without incurring adverse events.

## Introduction

Mitral regurgitation (MR) remains a condition strongly associated with adverse prognosis [1]. While transcatheter edge-to-edge repair has been increasingly adopted as a less invasive option, post-procedural recovery and in-hospital outcomes remain limited [2]. Therefore, mitral valve repair surgery continues to represent the most reliable treatment for durable correction of MR.

Mitral valve repair remains the gold-standard surgical treatment for mitral regurgitation. Among the various repair techniques, the loop method for artificial chordae (“neochordae”) reconstruction, routinely employed at our institution, is widely used [3–12]. In this method, chordal length is often determined by institutional preference or surgeon experience, which introduces variability in both operative technique and clinical outcomes. Typically, adjacent chordae presumed to be intact are used as reference points [5, 10]; however, these neighboring leaflets are frequently involved in pathological changes and may not be structurally normal. Additionally, variability in the size, depth, and insertion anatomy of papillary muscle chordae makes it unsuitable to directly apply the same chordal length from the contralateral papillary muscle. Loop-set anchoring usually defaults to the origin of the ruptured chordae; however, anatomical constraints or tissue fragility may render this region suboptimal.

We previously employed a strategy in which loop length was determined using chordae referenced from adjacent leaflets presumed intact and anchored the loop to the papillary muscle head corresponding to the ruptured chordae, typically on the occipital aspect in posterior leaflet prolapse. However, this method often resulted in residual leaflet billowing due to suboptimal chordal tension and recurrent mitral regurgitation related to inadequate papillary muscle support.

To address these limitations, we developed the RAFF method (Referring to the Anterior, Fixing on the Frontal), a novel approach to neochordae reconstruction designed to improve anatomical consistency and enhance surgical reproducibility.

This study aimed to evaluate the clinical utility of the RAFF strategy by comparing postoperative valve competence, procedural efficiency, and durability outcomes in individuals undergoing mitral valve repair.

## Materials and methods

### Study design, settings, and participants

This retrospective cohort study included all eligible individuals who underwent isolated, elective mitral valve repair at Gifu University Hospital between January 2019 and December 2024. Patients treated from January 2022 onward received the RAFF method, following its introduction under the surgical leadership of the senior author (TK). Cases before this date underwent mitral repair using the conventional loop technique. No formal randomization was performed; patient allocation was based on surgery date. Exclusion criteria included emergency operations, concomitant procedures other than tricuspid valve or arrhythmia surgery, and absence of informed consent. Follow-up data were obtained through routine outpatient visits and echocardiographic evaluations. Analyses were conducted on a complete-case basis, as no missing data were identified for either primary or secondary outcomes during the study period.

### Surgical procedure

**Fig. 1:**
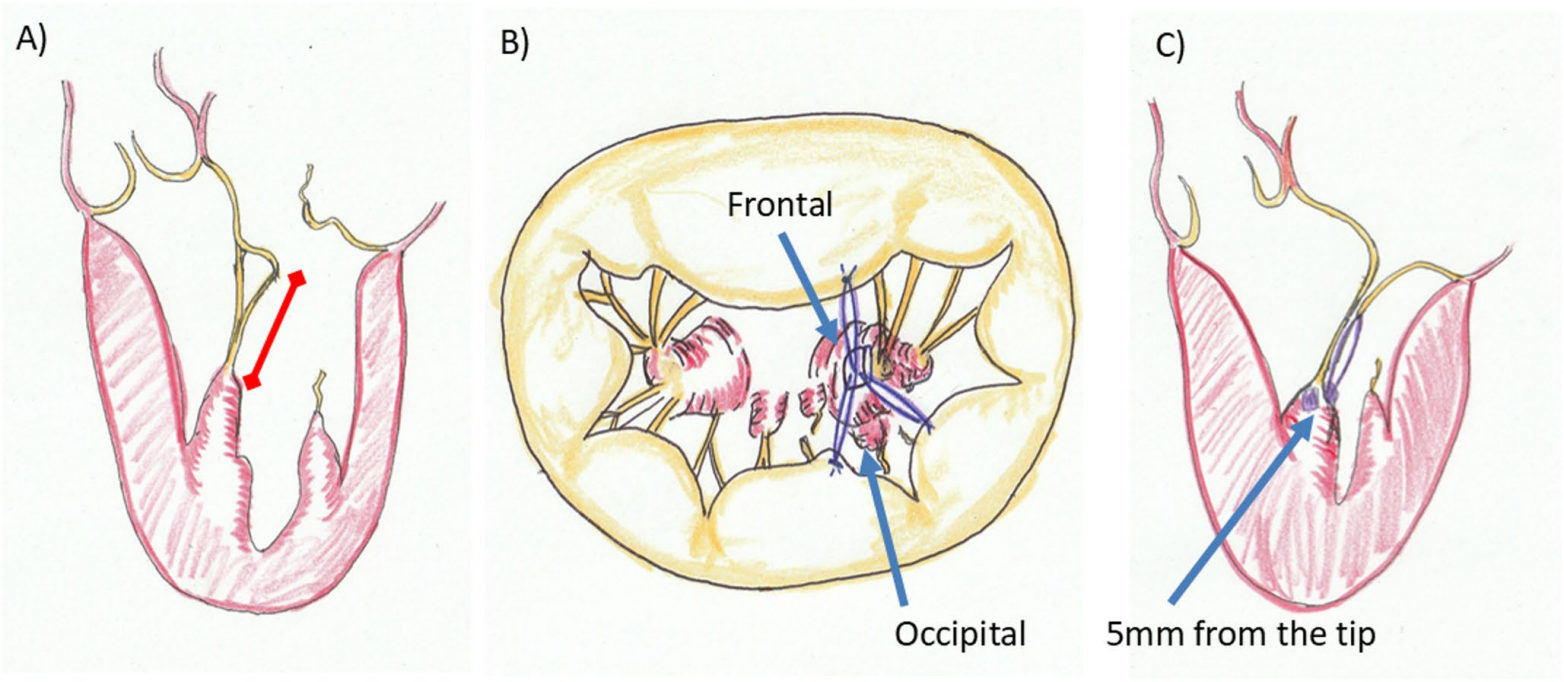
Conceptual illustration of the RAFF method. **a** Chordal length is determined using an anterior leaflet chord (or anterior strut chordae in cases of anterior or bileaflet prolapse, or Barlow’s disease) originating from the ipsilateral papillary muscle. This referencing method is applicable to posterior and commissural lesions. The bar indicates the reference chordal length. **b**, **c** The loop set is anchored 5 mm from the tip of the robust frontal aspect of the ipsilateral papillary muscle. All neochordae within the set are of equal length. In posterior leaflet prolapse, this anchoring results in anterior displacement of the posterior leaflet compared to conventional fixation at the occipital head, thereby increasing leaflet coaptation. This technique is referred to as “posterior neochordae frontal fixation.”

Mitral valve repair was performed via either standard median sternotomy or right mini-thoracotomy using a minimally invasive cardiac surgery (MICS) approach with endoscopic assistance. Cardiopulmonary bypass and cardioplegic arrest were applied according to standard protocols for each surgical route. To reduce potential bias, all procedures were performed or directly supervised by senior surgeons with 20 years of experience using a standardized operative protocol. Outcome assessments were conducted using uniform echocardiographic criteria across all participants. The annuloplasty ring or band size was selected based on the anterior leaflet area. The RAFF method is based on two principles (Fig. 1):

- Chordal length determination: Length is measured from an anterior leaflet chord (or strut chordae in cases of anterior, bileaflet prolapse, or Barlow’s disease) originating from the ipsilateral papillary muscle. This applies to posterior and commissural prolapse. For example, in a P3 lesion, the A2 medial chordae are used as a reference; for an anterolateral commissure lesion, the A2 lateral chordae are referenced.
- Papillary muscle head selection: The loop set is anchored 5 mm from the tip of the robust frontal head of the ipsilateral papillary muscle. Posterior leaflet chordae fixed to this frontal head are specifically described as “posterior neochordae frontal fixation.”

The technique was implemented as follows: (1) Chordal length was determined using the referenced anterior leaflet chordae; (2) loop sets were anchored 5 mm from the tip of the robust frontal head of the ipsilateral papillary muscle. Neochordae were created using expanded polytetrafluoroethylene (ePTFE) sutures (Gore-Tex CV4, W.L. Gore & Associates, Flagstaff, AZ, USA). Shibata’s technique was followed, utilizing a custom-designed workbench (Shibata Chordae System: 03-5425; Geister, Tuttlingen, Germany) [10].

In the non-RAFF group, chordal length was determined by referencing adjacent chordae presumed intact, and the loops were anchored to the corresponding papillary muscle head, typically the occipital head in posterior leaflet prolapse. For both groups, loops were secured to the rough zone of the prolapsed leaflet using CV5 sutures. No leaflet resection was performed. Intraoperative saline testing was used to evaluate residual mitral regurgitation and coaptation length. Fine-tuning, including neochordae refixation or edge-to-edge repair, was conducted when necessary. Valve competency was confirmed intraoperatively with transesophageal echocardiography (TEE), and re-repair or valve replacement was performed if residual dysfunction was detected.

Coaptation length, leaflet billowing, and residual MR grade were assessed using TEE at the end of surgery. MVPG and MR grade were assessed by transthoracic echocardiography (TTE) at discharge and at final follow-up.

### Statistical analysis

Continuous variables were summarized as medians with interquartile ranges (IQRs) and compared between the RAFF and non-RAFF groups using the Wilcoxon rank-sum test. Categorical variables were summarized as counts and percentages and compared using the Fisher exact test.

Binary outcomes were analyzed using multivariable logistic regression, adjusted a priori for surgical approach, concomitant procedures, and commissural lesions. Continuous outcomes were evaluated using linear regression with the same covariates. Analyses involving mitral valve peak gradient included additional adjustments for partial band annuloplasty and annuloplasty ring area indexed to body surface area ≥ 280 mm^2^, to control for known hemodynamic confounders.

Time-to-event outcomes were illustrated with Kaplan–Meier curves and compared using the log-rank test. Hazard ratios (HRs) were estimated with Cox proportional hazards models, adjusted for surgical approach, concomitant procedures, and commissural lesions. Ordinal outcomes were evaluated using ordinal logistic regression with the same covariate adjustments.

Coaptation length was compared between groups using the Wilcoxon rank-sum test.

All statistical tests were two-sided, and a *P* value < 0.05 was considered statistically significant. No adjustments were made for multiple comparisons, consistent with the exploratory nature of the study. All analyses were conducted using R version 4.3.2 (R Foundation for Statistical Computing, Vienna, Austria).

### Ethics statement

This investigation adhered to the principles of the Declaration of Helsinki [13]. Ethical approval was obtained from the Medical Ethics Committee of Gifu University Graduate School of Medicine, Japan (Record No.: 2024-319). All individuals provided written informed consent for participation and publication.

## Results

A total of 67 individuals were included, with a median age of 67 years and 67% male. The median follow-up duration was 936 days (range: 95–2246 days), with 100% follow-up completion. No missing data were identified for primary or secondary outcomes during the study period. The RAFF group (*n* = 36) and non-RAFF group (*n* = 31) demonstrated no significant differences in baseline characteristics (Table 1). Lesion distribution was comparable between the RAFF and non-RAFF groups: posterior leaflet (86% vs. 77%), anterior leaflet (22% vs. 32%), bileaflet (11% vs. 13%), and commissural lesions (33% vs. 35%). Use of partial annuloplasty rings was significantly higher in the RAFF group (72% vs. 32%, *p* = 0.01). No significant differences were observed in the number or length of neochordae, posterior chordal length, ring size distribution, or frequency of edge-to-edge repair. However, neochordae length was significantly longer in the RAFF group compared with the non-RAFF group (20.0 [16.0–20.0] mm vs. 16.0 [16.0–19.0] mm, *p* = 0.037). Rates of aortic cross-clamping for re-repair, conversion to valve replacement, and operative mortality were also similar. However, the RAFF group showed significantly less residual mitral regurgitation at the conclusion of surgery (*p* = 0.014) (Table 2).

**Table 1:** Patient characteristics

	Whole patients *N* = 67^a^	RAFF (−) *N* = 31^a^	RAFF (+) *N* = 36^a^	*p*-value^b^
Age	67.0 (56.0, 74.0)	68.0 (57.0, 72.5)	63.5 (54.8, 74.0)	0.734
Male	45 (67%)	21 (68%)	24 (67%)	> 0.999
Persistent atrial fibrillation	12 (18%)	7 (23%)	5 (14%)	0.355
Paroxysmal atrial fibrillation	6 (9%)	2 (6%)	4 (11%)	0.678
Chronic Kidney Disease	19 (28%)	10 (32%)	9 (25%)	0.592
*NYHA*				0.941
I	25 (37%)	11 (35%)	14 (39%)	
II	29 (43%)	15 (48%)	14 (39%)	
III	13 (19%)	5 (16%)	8 (22%)	
IV	0 (0%)	0 (0%)	0 (0%)	
Body Surface Area (m^2^)	1.6 (1.5, 1.8)	1.6 (1.5, 1.7)	1.6 (1.5, 1.8)	0.559
Asymptomatic	25 (37%)	10 (32%)	15 (42%)	0.459
Preoperative NT-proBNP (pg/ml)	260.0 (122.0, 799.5)	260.0 (114.0, 504.5)	256.5 (126.0, 951.0)	0.658
Preoperative LAd (mm)	43.0 (38.0, 47.0)	42.0 (38.0, 46.0)	44.0 (39.0, 47.3)	0.537
Preoperative LVDd (mm)	51.0 (47.0, 55.0)	51.0 (48.0, 54.0)	51.0 (46.0, 55.0)	0.724
Preoperative LVDs (mm)	31.0 (28.0, 33.8)	31.0 (28.5, 34.0)	30.0 (28.0, 33.5)	0.689
Preoperative LVEF (%)	65.0 (61.0, 70.0)	64.0 (59.5, 68.0)	68.0 (61.0, 72.0)	0.069
Preoperative TRPG (mmHg)	28.0 (21.0, 37.5)	27.0 (20.0, 31.5)	29.5 (22.0, 41.3)	0.054
Preoperative MR RV (ml)	82.0 (73.0, 108.0)	78.5 (68.5, 100.0)	91.0 (75.0, 110.5)	0.169

^a^Median (IQR); n (%)

^b^Wilcoxon rank sum test; Fisher’s exact test

**Table 2:** Operative findings

	Whole patients *N* = 67^a^	RAFF (-) *N* = 31^a^	RAFF (+) *N* = 36^a^	*p*-value^b^
Posterior leaflet lesion	55 (82%)	24 (77%)	31 (86%)	0.524
Anterior leaflet lesion	18 (27%)	10 (32%)	8 (22%)	0.415
Bi-leaflet lesion	8 (12%)	4 (13%)	4 (11%)	> 0.999
commissure leaflet lesion	23 (34%)	11 (35%)	12 (33%)	> 0.999
Height A2 (mm)	25.0 (23.0, 26.0)	25.0 (23.0, 26.0)	25.0 (23.3, 26.0)	0.847
Height P2 (mm)	18.0 (16.0, 20.0)	18.0 (16.0, 20.0)	18.0 (15.5, 20.0)	0.886
Number of neochordae	2.0 (2.0, 3.0)	2.0 (2.0, 3.0)	2.0 (2.0, 2.3)	0.332
Partial band annuloplasty	36 (54%)	10 (32%)	26 (72%)	0.001
Neochordae length (mm)	18.0(16.0, 20.0)	16.0(16.0, 19.0)	20.0(16.0, 20.0)	0.037
*Size of annuloplasty ring or band*				0.881
26	1 (2%)	0 (0%)	1 (3%)	
28	3 (5%)	0 (0%)	3 (9%)	
30	25 (38%)	15 (48%)	10 (29%)	
32	24 (36%)	10 (32%)	14 (40%)	
34	12 (18%)	6 (19%)	6 (17%)	
36	1 (2%)	0 (0%)	1 (3%)	
Edge-to-Edge repair	2 (3%)	2 (6%)	0 (0%)	0.21
Re-aortic cross clamp	8 (12%)	6 (19%)	2 (6%)	0.131
Conversion to replacement	2 (3%)	1 (3%)	1 (3%)	> 0.999
Operative death	0 (0%)	0 (0%)	0 (0%)	> 0.999
*Final residual MR at valvoplasty completion*				0.014
0, none	12 (18%)	3 (10%)	9 (25%)	
0.5, trivial-mild	45 (67%)	20 (65%)	25 (69%)	
1, mild	7 (10%)	6 (19%)	1 (3%)	
2, moderate	1 (1%)	1 (3%)	0 (0%)	
3, severe (conversion to replacement)	2 (3%)	1 (3%)	1 (3%)	

^a^Median (IQR); n (%)

^b^Wilcoxon rank sum test; Fisher’s exact test

Multivariable logistic regression was used to evaluate the association of the RAFF method with postoperative outcomes, adjusting for MICS approach, concomitant procedures, and commissural lesions. The RAFF method was significantly associated with reduced leaflet billowing and recurrence of moderate or greater mitral regurgitation (OR = 0.11, 95% CI 0.02–0.552, *p* = 0.012; OR = 0.14, 95% CI 0.02–0.71, *p* = 0.033, respectively). A trend toward reduced need for aortic cross-clamping during additional repair was observed in the RAFF group, though this did not reach statistical significance (OR = 0.17, *p* = 0.069). No significant differences were noted in rates of transient intraoperative systolic anterior motion (SAM), conversion to valve replacement, operative mortality, or postoperative complications (Table 3).

**Table 3:** Adjusted multivariable logistic regression analysis for RAFF method

	OR	95% CI	*p*-value
Transient SAM	6.15	0.79, 131	0.13
Leaflet billowing	0.11	0.02, 0.52	0.012
Re-aoritc cross clamp	0.17	0.02, 0.95	0.069
Failure of valvoplasty	0.94	0.03, 25.8	> 0.9
Operative death	1.00	0.00, Inf	> 0.9
Complications	0.32	0.06, 1.29	0.13
Recurrent MR ≥ moderate during follow-up	0.14	0.02, 0.71	0.033

Surgical approach, concomitant procedures, and commissural lesions were added to the model as covariates

Linear regression analysis was used to evaluate the association between the RAFF method and continuous outcomes (Table 4), adjusting for the MICS approach, concomitant procedures, commissural lesions, and, for pressure gradient analysis, use of partial band annuloplasty and annuloplasty ring area indexed to body surface area ≥ 280 mm². The RAFF group demonstrated significantly greater coaptation length (β = 1.7, 95% CI 0.99–2.4, *p* < 0.001). While cardiopulmonary bypass time did not differ between groups, aortic cross-clamp time was significantly shorter in the RAFF group (β = − 45 min, 95% CI − 71 to −  20, *p* < 0.001). The mean mitral valve gradient did not increase postoperatively in the RAFF group; instead, it was significantly lower at final follow-up (β = − 0.88, 95% CI − 1.5 to − 0.29, *p* = 0.004).

**Table 4:** Adjusted linear regression analysis for RAFF method

	Beta	95% CI	*p*-value
Coaptation length (mm)	1.7	0.99, 2.4	< 0.001
CPB time (min)	4.3	− 179, 187	> 0.9
Aortic cross clamp time (min)	− 45	− 71, − 20	< 0.001
Mean MVPG at discharge (mmHg)	0.03	− 0.48, 0.54	> 0.9
Mean MVPG at final follow-up (mmHg)	− 0.88	− 1.5, − 0.29	0.004

Surgical approach, concomitant procedures, and commissural lesions were added to the linear regression model as covariates for Coaptation length, CPB time, Aortic cross clamp time

Partial band annuloplasty and ring area/ BSA ≥ 280 mm² were added to the model as covariates for Mean MVPG at discharge and Mean MVPG at final follow-up

**Fig. 2:**
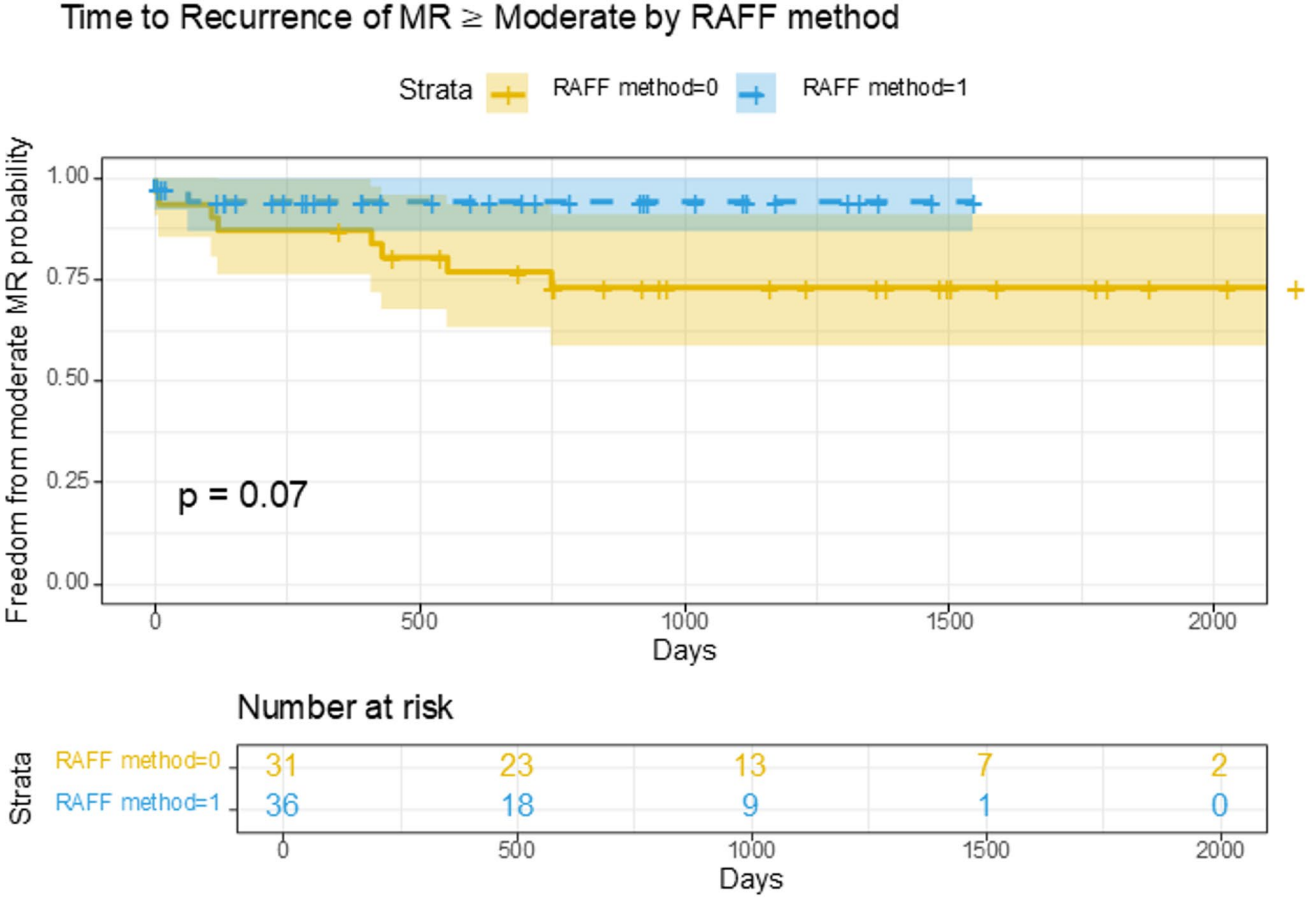
Kaplan–Meier analysis of freedom from moderate or greater mitral regurgitation stratified by the RAFF method. Patients who underwent mitral valve repair using the RAFF method (blue) demonstrated a trend toward greater freedom from recurrent mitral regurgitation compared to those treated without RAFF (yellow), although this difference did not reach statistical significance (*p* = 0.07)

Kaplan–Meier analysis demonstrated a trend toward reduced recurrence of moderate or greater mitral regurgitation in the RAFF group (*p* = 0.07) (Fig. 2), a finding supported by Cox proportional hazards modeling (HR = 0.26, 95% CI 0.06–1.24, *p* = 0.092)) (Table 5).

**Table 5:** Cox proportional hazards modeling for recurrent MR ≥ moderate

	HR	95% CI	*p*-value
RAFF method	0.26	0.06, 1.24	0.092

Ordinal logistic regression showed that the RAFF method significantly reduced mitral regurgitation grade at the end of surgery (Effect = 0.2, 95% CI 0.06–0.69, *p* = 0.0107), immediately postoperatively (Effect = 0.18, 95% CI 0.05–0.62, *p* = 0.0065), and at final follow-up (Effect = 0.11, 95% CI 0.03–0.38, *p* = 0.0004) (Table 6).

**Table 6:** Ordinal logistic regression analysis for postoperative MR grade

	aPORs	95% CI	*p*-value
MR grade at surgery completion	0.2	0.06, 0.69	0.0107
MR grade at discharge	0.18	0.05, 0.62	0.0065
MR grade at final follow-up	0.11	0.03, 0.38	0.0004

Concomitant procedures, MICS, and Commissural lesions were added to the ordinal logistic regression model as covariates

Subgroup analysis of the “posterior neochordae frontal fixation” technique revealed no significant increase in transient SAM (*p* = 0.2) or postoperative gradients (discharge: β = − 0.07, *p* = 0.8; final follow-up: β = − 0.61, *p* = 0.065). However, this technique was significantly associated with increased coaptation length (β = 1.7, 95% CI 0.94–2.4, *p* < 0.001). (Table 7)

**Table 7:** Sub-analysis (adjusted) for posterior neochordae frontal fixation

	OR	95% CI	*p*-value
Transient SAM	3.72	0.57, 34.7	0.2

	Beta	95% CI	*p*-value
Coaptation length (mm)	1.7	0.94, 2.4	< 0.001
Mean MVPG at discharge (mmHg)	− 0.07	− 0.55, 0.42	0.8
Mean MVPG at final follow-up (mmHg)	− 0.61	− 1.2, 0.04	0.065

The logistic regression model included concomitant procedures, MICS, and commissural lesions as covariates for Transient SAM. The linear regression model included concomitant procedures, MICS, and commissural lesions as covariates for Coaptation length. The linear regression model included partial band annuloplasty and ring area/BSA ≥ 280 mm² as covariates for Mean MVPG at discharge and Mean MVPG at final follow-up

## Comment

This study aimed to evaluate the clinical utility of the RAFF method in mitral valve repair. Our findings demonstrated that the RAFF technique significantly improved coaptation length, reduced residual mitral regurgitation, and shortened aortic cross-clamp time without increasing the risk of SAM or elevated transvalvular gradients. Accurate neochordae length determination is critical for durable mitral valve repair, and various strategies have been proposed to address this technical challenge. The loop technique, first described by Mohr et al., is known for procedural reproducibility and broad applicability, including in MICS settings [3, 6, 8, 9]. With regard to optimal neochordal length, Pitsis et al. demonstrated a strong correlation between preoperative transesophageal echocardiography (TEE) measurements and intraoperative chordal lengths [14]. Subsequent studies using preoperative CT or echocardiography have yielded similarly reliable correlations [14–18]. However, intraoperative measurements remain the gold standard.

In the RAFF method, chordal length is referenced from the anterior leaflet chordae arising from the papillary muscle ipsilateral to the prolapsing segment. In patients with bileaflet prolapse or Barlow’s disease, anterior strut chordae are used as the reference point [6, 9, 16, 19].

This strategy offers advantages over conventional techniques that rely on adjacent chordae near the prolapsing segment. The anterior leaflet is more likely to be anatomically intact, avoids inaccuracies caused by asymmetry between papillary muscles, and facilitates consistent measurement, even during MICS procedures.

For loop-set fixation, neochordae are anchored to the robust frontal aspect of the ipsilateral papillary muscle to minimize the risk of injury to fragile tissue. In the RAFF method, anchoring to the frontal head of the papillary muscle results in inherently longer neochordae, which is anatomically inevitable because the frontal head is located more anterior and distant compared to the occipital head.

The RAFF technique was associated with significantly greater coaptation length and suppression of residual leaflet billowing, without increasing the incidence of SAM or transvalvular gradient elevation. These improvements may contribute to the reduced recurrence of mitral regurgitation observed during follow-up. Although the Kaplan–Meier and Cox model results did not achieve statistical significance, they suggest clinically meaningful benefit and justify further validation.

Although adjustments were made for established predictors of postoperative occult mitral stenosis, such as an annuloplasty ring area/BSA < 280 [20], no adverse effects were observed. The significantly lower mean mitral gradient at final follow-up suggests favorable leaflet geometry and improved mitral mechanics under the RAFF method. Additionally, the shorter cross-clamp time likely reflects enhanced procedural efficiency through more consistent chordal length determination. These benefits are particularly advantageous in MICS contexts.

Initial concerns regarding SAM or elevated gradients following posterior leaflet frontal fixation were not substantiated, supporting the safety of this anchoring approach. To our knowledge, no previous studies have evaluated the potential benefits of avoiding the occipital papillary muscle head for neochordae fixation. This positions our findings as a novel contribution to current mitral valve repair strategies.

### Study limitations

This study represents a retrospective exploratory analysis with a limited sample size, which may introduce selection bias and limit the precision and generalizability of the findings. The absence of SAM in this cohort should also be interpreted with caution, as it is influenced by multiple factors and may not have been observed given the small number of cases.

The slightly higher mid- to long-term recurrence rate of mitral regurgitation observed in the non-RAFF group may partly reflect differences in surgical era and procedural consistency during the early phase of the study, rather than any intrinsic limitation of the RAFF approach.

Furthermore, because all procedures were conducted by experienced surgeons, the external applicability of the RAFF method to other clinical environments should be interpreted carefully. A future prospective study with a larger cohort is planned to validate and expand on these results and to confirm the long-term benefits of the RAFF technique, including its potential to reduce mitral regurgitation recurrence and improve overall patient outcomes.

## Conclusions

The RAFF method may offer a simplified, reproducible, and anatomically guided approach to neochordae reconstruction during mitral valve repair, optimizing leaflet geometry and supporting favorable valve function without increasing procedural risk. These retrospective findings suggest that the RAFF method may enhance surgical precision and repair durability. Prospective studies are warranted to confirm these outcomes across broader populations and surgical settings, and to determine its long-term benefits in comparison with other mitral valve repair methods. If validated in larger, multi-center studies, the RAFF method may provide a more standardized approach to mitral valve repair, reducing variability in surgical outcomes and improving long-term patient prognosis.

## Data Availability

The data underlying this article will be made available by the corresponding author upon reasonable request.
